# Exposure and Carriage of Pathogenic *Leptospira* in Livestock in St. Croix, U.S. Virgin Islands

**DOI:** 10.3390/tropicalmed6020085

**Published:** 2021-05-24

**Authors:** Hannah M. Cranford, Marissa Taylor, Andrew Springer Browne, David P. Alt, Tammy Anderson, Camila Hamond, Richard L. Hornsby, Karen LeCount, Linda Schlater, Tod Stuber, Leah De Wilde, Valicia J. Burke-France, Esther M. Ellis, Jarlath E. Nally, Bethany Bradford

**Affiliations:** 1 Virgin Islands Department of Health, Epidemiology Division, Virgin Islands, Christiansted, VI 00820, USA; hannah.cranford@doh.vi.gov (H.M.C.); Marissa.Taylor@doh.vi.gov (M.T.); springer.browne@vi.gov (A.S.B.); Leah.Dewilde@doh.vi.gov (L.D.W.); Valicia.BurkeFrance@doh.vi.gov (V.J.B.-F.); esther.ellis@doh.vi.gov (E.M.E.); 2 Domestic Animal Health Analytics Team, Animal and Plant Health Inspection Service (APHIS), United States Department of Agriculture, Fort Colins, CO 80526, USA; 3 National Centre for Animal Health *Leptospira* Working Group, United States Department of Agriculture, Ames, IA 50010, USA; david.alt@usda.gov (D.P.A.); tammy.m.anderson@usda.gov (T.A.); Camila.Hamond@usda.gov (C.H.); richard.hornsby@usda.gov (R.L.H.); karen.j.lecount@usda.gov (K.L.); linda.k.schlater@usda.gov (L.S.); tod.p.stuber@usda.gov (T.S.); jarlath.nally@usda.gov (J.E.N.); 4 Agricultural Research Service, Infectious Bacterial Diseases Research Unit, United States Department of Agriculture, Ames, IA 50010, USA; 5 National Veterinary Services Laboratories, Animal and Plant Health Inspection Service (APHIS), United States Department of Agriculture, Ames, IA 50010, USA; 6 U.S. Virgin Islands Department of Agriculture, Christiansted, VI 00820, USA

**Keywords:** leptospirosis, zoonoses, livestock, bacterial disease

## Abstract

From 2019–2020, the Virgin Islands Department of Health (VIDOH) investigated potential animal reservoirs of *Leptospira* spp., the pathogenic bacteria that cause leptospirosis. We examined *Leptospira* exposure and carriage in livestock on the island of St. Croix, United States Virgin Islands (USVI). We utilized the microscopic agglutination test (MAT) to evaluate the sera, and the fluorescent antibody test (FAT), real time polymerase chain reaction (rt-PCR), and bacterial culture to evaluate urine specimens from livestock (n = 126): 28 cattle, 19 goats, 46 pigs, and 33 sheep. Seropositivity was 37.6% (47/125) with agglutinating antibodies to the following serogroups identified: Australis, Djasiman, Icterohaemorrhagiae, Ballum, Sejroe, Cynopteri, Autumnalis, Hebdomadis, Pomona, Canicola, Grippotyphosa, and Pyrogenes. Urine from 4 animals (4.0%, 4/101) was positive by rt-PCR for lipL32: 2 sheep, 1 goat, and 1 bull. Sequencing of secY amplicons identified *L*. *interrogans* in 1 sheep and 1 bull. Livestock in USVI harbor pathogenic *Leptospira* bacteria and could play a role in the zoonotic cycle of leptospirosis.

## 1. Introduction

Pathogenic *Leptospira* species are diderm bacteria that cause the emerging infectious disease leptospirosis, a leading cause of global zoonotic disease with an estimated 1.03 million cases and 58,900 deaths annually worldwide [1]. *Leptospira* spp. are harbored in animal hosts and transmitted to humans via direct contact and environmental exposure to water or soil contaminated by infected animal urine. Persons who work with animals, including farmers, veterinary staff, and abattoir staff, may have occupational exposures to pathogenic *Leptospira* spp. [2]. In the United States, most cases have occurred in tropical and subtropical areas [3]. 

The United States Virgin Islands (USVI) is a territory of the United States of America, located in the Caribbean region, 40 miles east of Puerto Rico. USVI consists of three main islands, St. Croix, St. John, and St. Thomas, with a total land area of 133 square miles (344 square kilometers) and an estimated human population of 100,000. After the 2017 Hurricanes Irma and Maria, the Virgin Islands Department of Health (VIDOH) identified the first three cases of human leptospirosis in the USVI [4]. Two of three human cases were confirmed by microscopic agglutination testing (MAT) with the highest titer against *Leptospira interrogans* serovar (sv) Mankarso [4]. Before 2017, there was no active leptospirosis surveillance in the Territory. Cases likely went undiagnosed due to a lack of awareness, underreporting, and low healthcare seeking behaviors. A 2019 USVI cross-sectional serosurvey detected exposure to *Leptospira* serovars in an unweighted 3.9% of individuals sampled (n = 1206) [5]. Of 47 seropositive individuals, 51% (n = 24) had titers to two or more serovars [5]. The most common serogroups detected were Icterohaemorrhagiae, Australis, and Canicola (10 or more reactions) [5]. 

A previous research study in 1992 identified livestock exposed to *Leptospira* in St. Croix: goats (n = 28/108 positive) and sheep (n = 17/53 positive) were found reactive to eight *Leptospira* serogroups (Australis, Autumnalis, Ballum, Bataviae, Canicola, Icterohaemorrhagiae, Pyrogenes, and Sejroe) [6]. Similar investigations have been performed on other Caribbean islands such as Trinidad and Grenada. In Trinidad in 1985, a serosurvey of leptospirosis across livestock determined positivity in 92% of 26 cattle and 53% of 122 pigs [7]. A second study in 2010 determined positivity of 21.5% in cattle (n = 590), 5.0% in sheep (n = 222), 3.3% in goats (n = 180), and 5.0% in pigs (n = 200) [8]. In 1985, a similar survey in Grenada determined seropositivity to *Leptospira* spp. of 25% in 324 cattle, 35% in 130 pigs, 35% in 146 sheep, and 25% in 44 goats [7].

In August 2019, the VIDOH implemented an active public health surveillance system to identify sources of potential exposures and the possible reservoirs of leptospirosis in animals including rodents, mongooses, bats, dogs, and livestock. Surveillance of each species was concurrent. One isolate collected from a local mongoose was included in this investigation, as explained below. Here, we describe one part of the surveillance program: exposure and carriage of *Leptospira* spp. in livestock on St. Croix, USVI, using serology [microscopic agglutination test (MAT)], excretion of bacteria in urine [fluorescent antibody test (FAT) and lipL32 rt-PCR testing], and bacterial culture of *Leptospira*. 

## 2. Materials and Methods

We performed opportunistic sampling after animals were humanely processed in a USDA-approved abattoir. Historically, two abattoirs existed in USVI, one on St. Thomas and one on St. Croix. The 2017 hurricanes damaged the abattoir on St. Thomas, therefore all sampling was performed in St. Croix, where most USVI livestock are farmed. The processing of cattle, sheep, and pigs is performed throughout the year. There is no expectation of vaccination for leptospirosis for livestock in St. Croix [9]. We performed sampling one to four days per week from October 2019 to November 2020 except during abattoir closure from February 2020 to August 2020. All animals were processed at legally required ages for a USDA processing facility [9]. We collected blood via free-catch into a sterile cup during processing and transferred 3mL into a serum separator tube. We separated serum within 12 hours of collection and then stored samples in a −80 °C freezer until shipping. The serum was shipped in batches for MAT. We used sterile technique to collect urine (30 mL or as much as available, if less) by direct cystocentesis of the intact bladder using a 30 mL syringe. We performed cystocentesis immediately after evisceration, thus preventing cross-contamination by eliminating contact with the animal hide or facility surfaces. We inoculated culture media for *Leptospira* immediately with 3 drops of urine, while remaining urine was kept on ice after collection until processing. We shipped urine and inoculated *Leptospira* media overnight for culture, FAT, and rt-PCR. USDA Agricultural Research Service-National Animal Disease Center and National Veterinary Services Laboratories (Ames, IA, USA) tested all specimens.

For the detection of anti-*Leptospira* antibodies in the sera, we performed MAT according to World Organisation for Animal Health guidelines, using a panel of 18 antigens, representing 15 serogroups [10] (Appendix A). In addition, one locally recovered autochthonous strain isolated from a mongoose on St. Croix during public health surveillance activities was included in the MAT panel (species *Leptospira borgpetersenii*, serogroup Sejroe, sv undetermined). We defined a positive MAT as a titer equal to or greater than 1:100. 

We utilized culture, FAT, and rt-PCR to detect *Leptospira* spp. from livestock urine. For culture, we immediately inoculated semi-solid Hornsby-Alt-Nally (HAN) media containing 5-Fluoruracil (5-FU, 100 ug/mL) and semi-solid T80/40/LH media containing 5-Fluoruracil (5-FU, 100 ug/mL) with 3 drops of urine [11,12]. We shipped inoculated cultures overnight; upon arrival, we incubated T80/40/LH inoculated samples at 29 °C and HAN inoculated samples at 37 °C in 3% CO_2_. We determined specimens negative for bacterial culture if no growth was observed after six months. We performed FAT as previously described [13]. For rt-PCR, we extracted DNA from 30 mL of urine. We centrifuged the urine at 12,000 g for 30 min, rinsed the pellet once with 1 mL of PBS pH 7.4, then centrifuged it at 10,000 g for 15 min; we extracted total DNA with the Maxwell^®^ RSC Purefood Pathogen kit (Promega Corporation, Madison, WI, USA). We performed rt-PCR to detect the lipL32 gene as described [14,15]. We analyzed all samples in triplicate and considered a sample positive when duplicate or triplicate runs were positive with a cycle threshold (Ct) value <40.

For samples with positive lipL32 detection, we partially amplified the secY gene by PCR with primers secYF (5′-ATGCCGATCATTTTTGCTTC-3′) and secYR (5′-CCGTCCCTTAATTTTAGACTTCTTC-3′) followed by nested PCR with primers secYIVF (5′-GCGATTCAGTTTAATCCTGC-3′) and secYIVR (5′-GAGTTAGAGCTCAAATCTAAG-3′) as described previously [16,17,18]. We processed the resulting secY amplification reactions using QIAquick 96 PCR purification kits (Qiagen, Hilden, Germany) according to manufacturer’s directions. We quantified resulting material with the Qubit™ dsDNA BR assay kit (ThermoFisher Scientific, Waltham, MA, USA) using a Qubit 2.0 fluorometer (ThermoFisher Scientific, Waltham, MA, USA). We labeled amplicons using the Applied Biosystems BigDye™ Terminator v3.1 Cycle Sequencing Kit and precipitated and suspended them for sequencing using an Applied Biosystems 3130xl Genetic Analyzer according to manufacturer’s directions (Applied Biosystems, Foster City, CA, USA). We performed analysis using R (package version 3.5.0) [19].

## 3. Results

We sampled 126 livestock from 33 farms from the island of St. Croix, USVI. Table 1 describes animals sampled.

We observed the overall livestock seropositivity to be 37.6% (95% CI 29.1–46.7, n = 47/125). Nineteen animals had antibodies to more than one serovar. Of 90 reactions observed (Appendix B), the most reactive serogroups are as follows: Australis (33.3%), Djasiman (16.7%), Icterohaemorrhagiae (15.6%), Ballum (10.0%), Sejroe (9.0%), Cynopteri (5.6%), Autumnalis (2.2%), Hebdomadis (2.2%), Pomona (2.2%), Canicola (1.1%), Grippotyphosa (1.1%), and Pyrogenes (1.1%). Two livestock samples collected from a sheep and a pig from separate farms had exceptionally high titers, respectively: 1:6400 against Icterohaemorrhagiae (LL110) and 1:12800 against Sejroe (LL120). Of 32 MAT positive pigs, 26 displayed reactivity to Australis (sv Bratislava). One serum sample was not tested due to insufficient volume. Table 2 shows titer reactivity by serogroup and by sampled species. 

**Figure 1:**
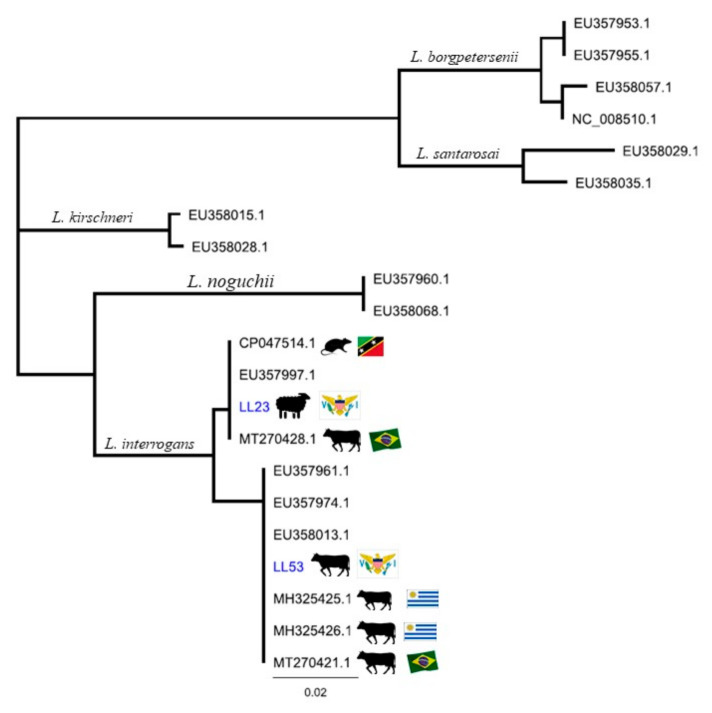
Phylogeny of *Leptospira* spp. based on secY gene sequence analysis. Neighbor-Joining method. The evolutionary distances were computed using the Tamura-Nei method. St. Croix, U.S. Virgin Islands clinical samples (LL23; LL53) are in blue.

Of 101 urine samples, 4 tested positive by rt-PCR for lipL32: 2 sheep (LL23 and LL60, which had Ct values of 34.5 and 38.2, respectively), 1 goat (LL36, Ct 37.8), and 1 bull (LL53, Ct 32). Two of these samples yielded secY sequences, LL23 and LL53, with 100% of identity to *Leptospira interrogans*. Phylogeny based on secY IV gene sequence analysis revealed that LL53 cluster together with *L. interrogans* sv Canicola (MH325426.1) and Pomona (MH325425.1) isolated from cattle urine in Uruguay, and *L. interrogans* (MT270421.1) from cattle urine sample in Brazil, as well as with a reference strain *L. interrogans* sv Canicola and *L. interrogans* sv Pomona [20,21]. However, LL23 aligns more closely with *L. interrogans* serogroup Icterohaemorrhagiae strain R19 (CP047514.1) isolated from a rodent in Saint Kitts and from cattle urine (MT270428.1) from Brazil and with reference strains [20,22] (Figure 1). These findings, along with 3 SNPs between the secY sequences (Appendix C), suggest that the sheep and bull are shedding distinct *Leptospira* bacteria. Both LL23 and LL53 showed no exposure to leptospirosis (i.e., both serum samples were MAT negative), which highlights the limitation of using serology to infer renal carriage of *Leptospira* spp., as well as the unique biological equilibrium that exists between pathogen and host. 

Of 97 urine samples, none were FAT positive, and we did not successfully culture any *Leptospira* isolates. Table 3 displays livestock sample results by species.

## 4. Discussion

Evidence of *Leptospira* exposure (MAT) and carriage (rt-PCR) is evident in livestock on St. Croix, USVI. Although seropositivity rates in goats and sheep in St. Croix dropped by half from 1992 to 2019–2020, the seroprevalence of 37.6% found in this study is similar to that found in studies from regions with similar topography and tropical conditions as USVI which allows for environmental prevalence and transmission of *Leptospira* spp. [6,23]. Furthermore, this investigation is likely a more robust evaluation of all farms on St. Croix, as we sampled animals from 33 farms over a year, while Ahl et al. sampled one sheep herd and eight goat herds [6]. 

Further comparison of these data with the 1992 Ahl et al. study on St. Croix, shows overlapping detection of four serogroups (Australis, Autumnalis, Ballum, and Sejroe). Similarly, when evaluating these data against the 1985 and 2010 surveys of livestock on the Caribbean islands of Trinidad and Grenada, similar *Leptospira* serogroups were detected: three in Trinidad in 1985 (Autumnalis, Hebdomadis, and Icterohaemorrhagiae); four in Grenada in 1985 (Autumnalis, Icterohaemorrhagiae, Hebdomadis, and Sejroe); and five in Trinidad in 2010 (Autumnalis, Bratislava, Hardjo, Icterohaemorrhagiae, and Sejroe) [7,8]. These serogroups appear to be prevalent throughout the Caribbean region, although cross-reactivity on MAT is common.

Notably, we observed a seropositivity of 67% (32/46) in St. Croix pigs, with most high titer reactions to serogroups Australis, Djasiman, and Icterohaemorrhagiae. In 1985, Everard et al. found that 35% (41/130) of Grenadian pigs were seropositive, of which 35% were reactive to serogroup Autumnalis and 32% to Icterohaemorrhagiae. The same study also reported that of Trinidadian pigs, 52% (64/122) were seropositive with 29% reactive to serogroup Autumnalis and 56% reactive to serogroup Icterohaemorrhagiae [7]. However, there was a sharp decline in seropositivity in Trinidadian pigs from 52% in 1985 to 5% in Suepaul et al.’s study in 2011, which mirrors the decline in seropositivity of St. Croix goats and sheep mentioned above [6,7,8].

Of the four animals that were PCR positive for lipL32, the two with the lowest Ct values were also positive by nested-PCR for secY. Sequencing of secY amplicons indicates that both animals were positive for *L. interrogans*. Interestingly, and as observed with other livestock, the positive PCR urine samples were obtained from animals that were seronegative for all serovars tested [24,25]. We were not able to definitively identify the serovar involved since we did not isolate the bacteria using culture methods.

In the USVI, livestock farming is low intensity as most farms have few animals. There are approximately 135 farms on St. Croix, 40 on St. Thomas, and 15 on St. John, with registered livestock totals for the Territory of 1700 pigs, 1200 cattle, 5000 goats, and 5400 sheep [9]. Due to the low intensity of farming on St. Croix, with closer contact between farmers, their families, and their stock, there is a higher risk of exposure to urine of infected animals compared to commercial operations. Generally, with pigs, there is a higher frequency of contact and care compared to grazing animals (ruminants), providing farmers and abattoir-workers a greater risk of contact with *Leptospira* spp. Concern for public health regarding ruminants may be even lower considering most USVI cattle (Senepol breed, developed on St. Croix, USVI) are bred for meat, not dairy, which would mitigate potential exposures from animal to human from daily milking [26].

Following the detection of the first three cases of human leptospirosis in USVI after the 2017 Hurricanes Irma and Maria, VIDOH identified objectives: strengthen the local leptospirosis surveillance program, identify sources of exposure to *Leptospira* spp., and expand provider knowledge and public health education regarding the infection. This survey of livestock on St. Croix, USVI, reinforces previous findings surrounding livestock carriage and shedding of *Leptospira* spp. and adds to the limited available data on *Leptospira* reservoirs in the Caribbean region. Conducting this survey allowed for VIDOH staff to provide education to agricultural workers on the risk of occupational exposure, practices to mitigate risk, and signs and symptoms of illness. The results of this survey will inform farmers and other persons who work with USVI livestock on the potential for exposure to infectious pathogens. This project built long-lasting leptospirosis prevention and surveillance capacity within the USVI by supplying essential equipment and training to local staff, building a laboratory network for more robust testing resources, and engaging diverse stakeholders unified towards leptospirosis programming. Lastly, the results from this survey highlight the importance of diverse laboratorial testing for *Leptospira* spp. to maintain robust disease surveillance, such as inclusion of FAT, rt-PCR, and culture. In conclusion, livestock in St. Croix, USVI, are exposed to and harbor pathogenic *Leptospira* spp., which could play a role in the risk of exposure and transmission of leptospirosis infection from animals to humans. 

## Figures and Tables

**Table 1:** Characteristics of livestock sampled (n = 126) for leptospirosis in St. Croix, U.S. Virgin Islands.

Species	Female	Male	Total Animals	Number of Farms
Cattle	17	11	28	5
Goat	19	0	19	8
Pig	25	21	46	13
Sheep	7	26	33	12
Total	49	77	126	33 ^†^

^†^ 33 unique farms represented, some farms had more than one species processed.

**Table 2:** Microscopic agglutination test (MAT) results by livestock species and serogroup detected and the associated confidence intervals ^†^.

Livestock Sampled	Serogroup	%	95% CI
Cattle (n = 28)	Ballum	10.7% (3/28)	2.3–28.2
	Sejroe	10.7% (3/28)	2.3–28.2
	Icterohaemorrhagiae	7.1% (2/28)	0.8–23.5
	Australis	3.6% (1/28)	0.1–18.4
	Djasiman	3.6% (1/28)	0.1–18.4
Goat (n = 19)	Cynopteri	5.3% (1/19)	0.1–26.0
	Icterohaemorrhagiae	5.3% (1/19)	0.1–26.0
Pig (n = 46)	Australis	56.5% (26/46)	41.1–71.1
	Djasiman	28.3% (13/46)	16.0–43.5
	Icterohaemorrhagiae	21.7% (10/46)	11.0–36.4
	Ballum	15.4% (4/46)	2.4–10.8
	Sejroe	15.4% (4/46)	2.4–10.8
	Cynopteri	6.5% (3/46)	1.4–17.9
	Grippotyphosa	2.2% (1/46)	0.1–11.5
	Hebdomadis	2.2% (1/46)	0.1–11.5
	Pomona	2.2% (1/46)	0.1–11.5
Sheep (n = 33)	Australis	9.1% (3/33)	1.9–24.3
	Autumnalis	6.1% (2/33)	0.7–20.2
	Ballum	6.1% (2/33)	0.7–20.2
	Canicola	3.0% (1/33)	0.1–15.8
	Cynopteri	3.0% (1/33)	0.1–15.8
	Djasiman	3.0% (1/33)	0.1–15.8
	Sejroe	3.0% (1/33)	0.1–15.8
	Hebdomadis	3.0% (1/33)	0.1–15.8
	Icterohaemorrhagiae	3.0% (1/33)	0.1–15.8
	Pomona	3.0% (1/33)	0.1–15.8

^†^ More than one serogroup was detected.

**Table 3:** Results (positive/total) of livestock serum and urine samples tested using MAT, FAT, rt-PCR and culture by animal species in St. Croix, U.S. Virgin Islands ^†^.

Species	MAT	FAT	rt-PCR	Culture
Cattle	7/28 (25.0%, 95% CI 10.7–45.0)	0/28	1/28 (3.6%, 95% CI 0.1–18.4)	0/28
Goat	2/18 (11.1%, 95% CI 1.4–34.7)	0/16	1/16 (6.3%, 95% CI 0.2–30.2)	0/16
Pig	32/46 (67.0%, 95% CI 54.3–82.3)	0/22	0/25 (0%)	0/27
Sheep	6/33 (18.2%, 95% CI 7.0–35.5)	0/31	2/32 (6.3%, 95% CI 0.8–21.0)	0/32
Totals	47/125 (37.6%, 95% CI 29.1–46.7)	0/97	4/101 (4.0%, 95% CI 1.1–10.0)	0/103

^†^ FAT: florescent antibody test; rt-PCR: real time polymerase chain reaction; MAT: microscopic agglutination test.

## Data Availability

The data presented in this study are available in Appendix A, Appendix B, and Appendix C.

## Appendix A

**Table A1:** *Leptospira* serovars and serogroups in the microscopic agglutination testing (MAT) panel used by USDA-ARS-NADC in the testing of livestock serum samples in St. Croix, U.S. Virgin Islands.

		Serogroup	Serovar
1.		Australis	Australis
2.		Australis	Bratislava
3.		Autumnalis	Autumnalis
4.		Ballum	Ballum
5.		Bataviae	Bataviae
6.		Canicola	Canicola
7.		Cynopteri	Cynopteri
8.		Djasiman	Djasiman
9.		Grippotyphosa	Grippotyphosa
10.		Hebdomadis	Hebdomadis
11.		Icterohaemorrhagiae	Copenhageni
12.		Mini	Szwajizak
13.		Pomona	Pomona
14.		Pyrogenes	Pyrogenes
15.		Sejroe	Hardjo
16.		Sejroe	Undetermined (LM31) ^†^
17.		Sejroe	Sejroe
18.		Tarassovi	Tarassovi

^†^ Autochthonous strain recovered from a mongoose on St. Croix, USVI, species *Leptospira borgpetersenii*, serogroup Sejroe, serovar undetermined.

## Appendix B

**Table A2:** Titer and associated serovar(s) (n=47/125) of microscopic agglutination test (MAT) positive livestock serum samples in St. Croix, U.S. Virgin Islands.

Specie	#	AUS ^†^	AUT	BAL	BAT	BRA	CAN	CYN	DJA	GRI	HAR	HEB	ICT	MIN	POM	PYR	SEJ	TAR	LM31
**Cattle**	**51**										**100**								
	**54**					**100**													
	**69**			**100**															
	**82**			**100**					**100**										
	**83**												**200**						
	**103**										**100**								
	**117**			**100**							**100**		**100**						
																			
**Goat**	**49**							**100**											
	**100**												**200**						
																			
**Pig**	**56**								**100**										
	**57**					**100**													
	**61**					**200**			**200**										
	**63**					**100**													
	**65**								**100**										
	**66**					**200**			**100**										
	**67**			**100**															
	**70**					**400**		**100**	**200**				**100**						
	**71**					**100**													
	**72**					**400**			**100**										
	**75**					**400**													
	**78**					**200**			**200**										
	**80**					**100**							**400**						
	**81**			**400**		**400**							**200**						
	**85**					**400**							**100**						
	**87**					**400**													
	**88**					**100**													
	**90**					**200**													
	**91**												**200**						
	**92**					**400**													
	**93**					**200**													
	**104**					**400**			**100**								**100**		
	**106**					**200**													
	**107**								**100**										
	**108**					**100**			**100**										
	**109**			**100**		**400**		**200**	**800**	**100**			**200**						
	**120**					**800**					**3200**	**800**	**100**				**12800**		**3200**
	**121**					**1600**		**100**	**100**				**400**						
	**123**								**200**										
	**124**					**200**							**400**						
	**125**					**100**													
	**126**			**100**		**400**							**100**		**100**				
																			
**Sheep**	**45**					**100**													
	**47**		**100**			**100**													
	**48**							**100**											
	**59**											**200**							
	**102**			**100**															
	**110**		**100**	**800**		**800**	**3200**		**200**		**100**		**6400**		**100**	**800**			

^†^ AUS = Australis, serovar (sv) Australis; AUT = Autumnalis, sv Autumnalis; BAL = Ballum, sv Ballum; BAT = Bataviae, sv Bataviae; BRA = Australis, sv Bratislava; CAN = Canicola, sv Canicola; CYN = Cynopteri, sv Cynopteri; DJA = Djasiman, sv Djasiman; GRI = Grippotyphosa, sv Grippotyphosa; HAR = Sejroe, sv Hardjo; HEB = Hebdomadis, sv Hebdomadis; ICT = Icterohaemorrhagiae, sv Copenhageni; MIN = Mini, sv Szwajizak; POM = Pomona, sv Pomana; PYR = Pyrogenes, sv Pyrogenes; SEJ = Sejroe, sv Sejroe; TAR = Tarassovi, sv Tarassovi; LM31 = Autochthonous strain recovered from a mongoose on St. Croix, USVI, species *Leptospira borgpetersenii*, serogroup Sejroe, serovar undetermined.

## Appendix C. secY IV Sequence for Livestock Urine Samples in St. Croix, U.S. Virgin Islands, with SNPs Highlighted in Yellow ^†^

LL23 AGATTGGAGTTAGAGCTCAAATCTAAGAATTTTATAATAATATAAGGTGCTAATGCCAAA	60
LL53 ------GAGTTAGAGCTCAAATCTAAGAATTTTATAATAATATAAGGTGCTAATGCCAAA	54

LL23 CCTGCAAGAAACATAGCTCCGGGAAGAGTGATTCTATTTAACACTTTTTCAATGTATTCT	120
LL53 CCTGCAAGAAACATAGCTCCAGGGAGAGTGATTCTATTTAACACTTTTTCAATGTATTCT	114

LL23 TTTGTGTGAGAACCCGGACGAATTCCTGGAATGAACCCACCGTATTTTTTCAAATTCTCA	180
LL53 TTTGTGTGAGAACCCGGACGAATTCCTGGAATGAATCCACCGTATTTTTTCAAATTCTCA	174

LL23 GCCAATTCTGCAGGATTAAACTGAATCGCAATAC	214
LL53 GCCAAT----------------------------				180

^†^ Autochthonous strain recovered from a mongoose on St. Croix, USVI, species *Leptospira borgpetersenii*, serogroup Sejroe, serovar undetermined.
